# Henry’s Solubility and Diffusion Coefficients for 29 Volatile Organic Compounds in Polydimethylsiloxane Sylgard 184 at 293 K

**DOI:** 10.1021/acsomega.6c02987

**Published:** 2026-07-10

**Authors:** Cody B. Cockreham, Brandon L. Foley, Fang Qian, Maxwell Murialdo

**Affiliations:** Materials Science Division, 4578 Lawrence Livermore National Laboratory, 7000 East Ave, Livermore, California 94550, United States

## Abstract

Two-dimensional (2D) inverse gas chromatography (IGC) enables simultaneous determination of Henry’s solubility and Fickian diffusion coefficients for volatile organic compounds (VOCs) in polymer films. This technique offers a significant advantage over traditional cylindrical column IGC by providing precise control and measurement of the film thickness (here, 0.064 ± 0.002 mm), which is the critical length scale for accurate diffusivity determination. We apply this methodology to characterize VOC transport in Sylgard 184, a widely used polydimethylsiloxane (PDMS)-based polymer containing substantial silica filler content. At room temperature (20 °C), we measured solubility and diffusion coefficients for 29 common VOCs spanning diverse chemical functionalities, including alkanes, aromatics, chlorinated solvents, ketones, esters, and alcohols. Comparison with literature data for pure PDMS reveals that VOC solubility in Sylgard 184 is generally higher; for most non-hydrogen-bonding compounds it remains within a factor of 2 of pure PDMS, whereas alcohols are enhanced by roughly 1.8 to 3.7 times, which we attribute to favorable interactions with residual silanol groups on the silanized silica filler. Diffusion coefficients range from 1.0 × 10^–6^ cm^2^/s (n-undecane) to 8.9 × 10^–5^ cm^2^/s (acetonitrile) and align well with extrapolated literature values for PDMS. This study provides essential thermodynamic and transport data for predicting VOC permeation in Sylgard 184 while demonstrating the utility of 2D IGC as a robust technique for characterizing rubbery polymer membranes across diverse industrial applications.

## Graphical abstract

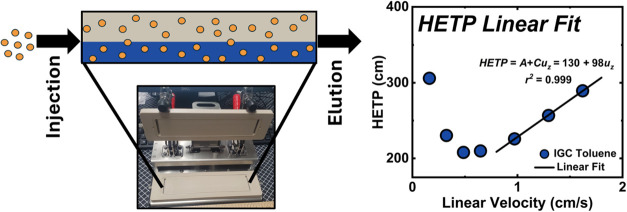

## Introduction

Polymer materials encounter volatile organic compounds (VOCs) throughout their service lifetime from multiple sources: processing solvents, outgassing products during thermal aging, and biologically generated organics in environmental exposures. VOCs are organic chemicals with high vapor pressures at ambient temperature that readily partition into polymers, potentially causing swelling, plasticization, degradation, or other deleterious aging effects.^1–4^ Understanding VOC-polymer interactions requires quantification of two fundamental transport properties: the equilibrium solubility of the vapor in the polymer matrix and the rate at which vapor molecules diffuse through the polymer network. For dilute vapor systems (low partial pressures), these properties are captured by two parameters: Henry’s solubility coefficient, which describes absorption equilibrium at infinite dilution, and Fickian diffusion coefficient, which quantifies the concentration-driven mass flux according to Fick’s laws. Together, these coefficients enable thermodynamic and kinetic predictions for vapor-polymer systems across diverse applications.

Inverse gas chromatography (IGC) has emerged as a powerful technique for determining vapor-polymer equilibria and transport properties. Unlike conventional gas chromatography where the stationary phase is used to separate analytes, IGC employs the material of interest as the stationary phase while systematically varying the injected probe vapors.^5^ By measuring retention times of vapor pulses through a polymer-packed column, Henry’s solubility coefficients can be directly extracted. Beyond equilibrium measurements, IGC simultaneously yields diffusion coefficients through analysis of peak broadening using the van Deemter equation, which relates theoretical plate height to various mass transfer resistances in the column.^6^ This dual capability makes IGC exceptionally efficient for comprehensive polymer characterization.

Traditional IGC employs cylindrical columns packed with polymer-coated support particles (typically surface-treated silica beads). The polymer coating thickness on these particles must be estimated and subtracted from the substrate contribution during data analysis. Because these diffusivity calculations depend on the square of the relevant length scale, uncertainties in coating thickness propagate significantly into calculated diffusivity values. Moreover, ensuring coating uniformity across all particles presents practical challenges. A more direct approach utilizes two-dimensional (2D) columns containing polymer thin films, where film thickness can be precisely measured and verified.^7^ While a few pioneering studies have explored film-based IGC for solubility and diffusivity measurements,^8,9^ this approach remains underutilized despite its advantages.

For this study, we selected Sylgard 184 as a commercially relevant and scientifically interesting polymer system. Sylgard 184 is predominantly composed of cross-linked polydimethylsiloxane (PDMS) with substantial silica filler content (30–60 wt %). PDMS itself is extensively studied, with numerous IGC investigations of VOC solubility and diffusivity across various temperatures and polymer formulations.^10–15^ As a soft, rubbery silicone elastomer, PDMS has achieved widespread adoption across biomedical engineering, additive manufacturing, microfluidic devices, flexible electronics, and soft robotics.^16–21^ This prevalence stems from PDMS’s favorable combination of thermal and chemical stability, mechanical flexibility, tunable optical properties, and low surface energy.^22–26^ Sylgard 184 from Dow Chemical represents one of the most widely deployed commercial PDMS formulations. However, its composite nature (featuring dimethylvinylated and trimethylated silica fillers intended to modify mechanical properties) may lead to sorption and transport behavior distinct from pure PDMS chains.^27,28^ The silica surface treatment with hexamethyldisilazane reduces hydrophilicity and improves dispersion within the PDMS matrix, though complete surface modification is unlikely, potentially leaving residual silanol (Si–OH) groups capable of hydrogen bonding.

This study advances the application of 2D IGC for polymer characterization while providing comprehensive VOC transport data for Sylgard 184. We report Henry’s solubility and Fickian diffusion coefficients for 29 VOCs at 20 °C and establish experimental protocols for extracting these properties from 2D IGC measurements in thin-film geometries.

## Experimental Section

### Sample Preparation

Sylgard 184 silicone elastomer kit was obtained from Dow Corning and used as received. The base and cure components were thoroughly mixed in a 10:1 weight ratio, followed by degassing in a vacuum desiccator, then spin-coated onto a silicon wafer at 1500 rpm for 30 s and cured at 80 °C for 1 h. The film was measured to be 0.064 ± 0.002 mm thick, where the reported uncertainty is the standard deviation of five measurements taken at different locations across the film. The cured films were carefully peeled off before testing. Uniformity was verified using a thickness measurement gauge while maintaining a contact force resistance below 0.1 N. Although the exact composition is proprietary, the composition has been reported to contain dimethylsiloxane oligomers with vinyl-terminated end groups (>60%), silica filler (dimethylvinylated and trimethylated silica, 30–60 wt %), tetra­(trimethylsiloxy)­silane (1–5%) and ethylbenzene (<1%) in the base and a cross-linking agent (dimethylmethylhydrogensiloxane, 40–70%) and an inhibitor (tetramethyltetravinylcyclotetrasiloxane 1–5%) in the curing agent.^29^

### Inverse Gas Chromatography Equipment and Preparation

Inverse gas chromatography (IGC) experiments were conducted using Surface Measurement Systems Ltd.’s Inverse Gas Chromatography-Surface Energy Analyzer (iGC-SEA). Pulses of organic vapor through a column containing the sample material (thin film of Sylgard 184) using a mass flow controller and ultrahigh-purity (UHP, 99.999%) nitrogen as a carrier gas. Details of the solvents and gases used in these experiments are provided in Table 1. Measurements were conducted using a two-dimensional (2D) column provided by Surface Measurement Systems Ltd. A schematic of the 2D style rectangular column is presented in Figure 1a, and an image of the column is presented alongside the schematic in Figure 1b. The interior column dimensions were measured to be 177 × 40 × 1.35 mm^3^ (length × width × height). The 2D column does not have active thermal control. Room temperature was measured to be 20 °C using a thermometer. Vapors are detected using a flame ionization detector fueled by hydrogen and compressed air. Before measurement the film was pretreated for 3 h under 50 cc/min flow of nitrogen. Saturation pressures at temperatures are determined from constants extracted from Perry’s Chemical Engineering Handbook ninth Edition.^30^

**Figure 1:**
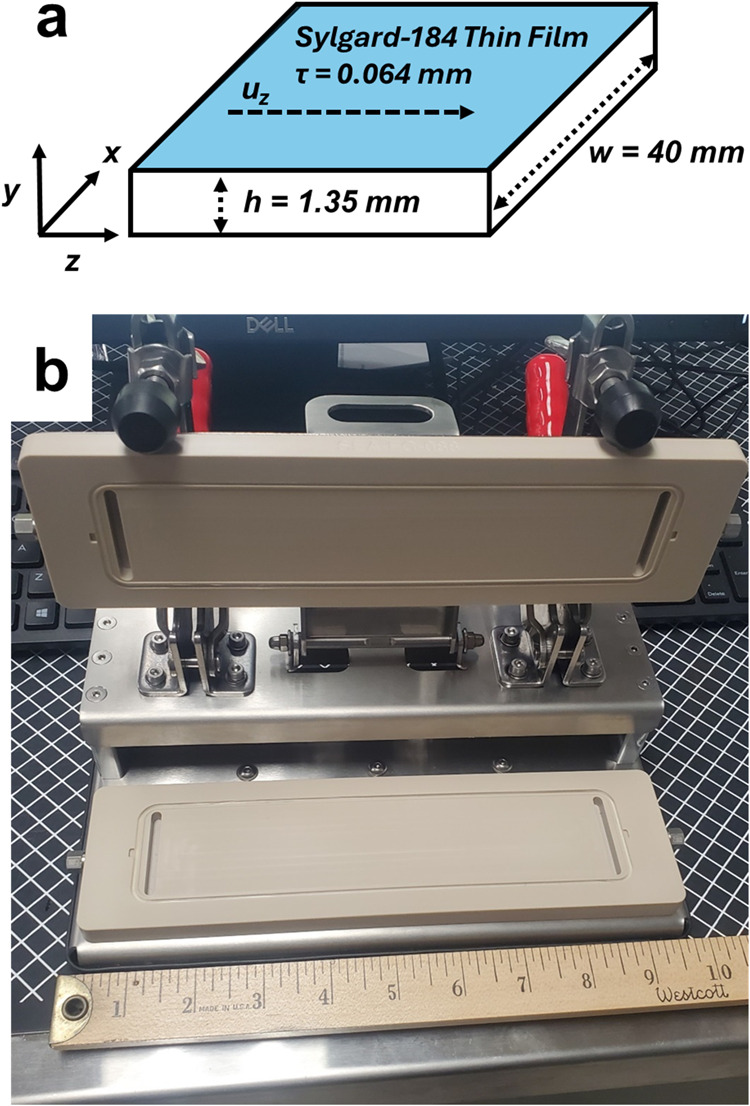
(a) Schematic of the two-dimensional (2D) column used in inverse gas chromatography (IGC) measurements. Dimensions are presented as height of the column (*h*), width of the column (*w*), and the length of the column (*z*). The flow direction of the gas is denoted by linear velocity (*u*_*z*_). (b) Picture of the 2D column used in IGC measurements of the thin film.

**Table 1:** Detailed Information on the Volatile Organic Compounds and Gas Sources

chemical name	CAS No.	source	product No.	purity (%)
*n*-hexane	110–54–3	Sigma-Aldrich	139386	≥99
2,3-dimethylbutane	79–29–8	Sigma-Aldrich	D151602	98
2-methylpentane	107–83–5	Thermo Scientific Chemicals	127340025	>99
*n*-heptane	142–82–5	Sigma-Aldrich	34873	≥99
*n*-octane	111–65–9	Sigma-Aldrich	296988	≥99
isooctane	540–84–1	Sigma-Aldrich	1.1544	≥99.8
*n*-nonane	111–84–2	Sigma-Aldrich	296821	≥99
*n*-decane	124–18–5	Sigma-Aldrich	457116	≥99
n-undecane	1120–21–4	TCI America	U0002	>99
cyclohexane	110–82–7	Sigma-Aldrich	650455	≥99.7
cyclooctane	292–64–8	Sigma-Aldrich	C109401	≥99
toluene	108–88–3	Sigma-Aldrich	650579	≥99.9
o-xylene	95–47–6	Sigma-Aldrich	95662	≥99.0
dichloromethane	75–09–2	Sigma-Aldrich	650463	≥99.9
chloroform	67–66–3	Sigma-Aldrich	650498	≥99.9
carbon tetrachloride	56–23–5	Sigma-Aldrich	270652	≥99.9
acetonitrile	75–05–8	Sigma-Aldrich	34998	≥99.9
diethyl ether	60–29–7	Sigma-Aldrich	309966	≥99.9
acetone	67–64–1	Sigma-Aldrich	270725	≥99.9
methylethyl ketone	78–93–3	Sigma-Aldrich	34861	≥99.7
ethyl acetate	141–78–6	Sigma-Aldrich	650528	≥99.9
butyl acetate	123–86–4	Sigma-Aldrich	270687	≥99.7
methanol	67–56–1	Sigma-Aldrich	1.06018	≥99.8
ethanol	64–17–5	Sigma-Aldrich	459844	≥99.5
1-propanol	71–23–8	Sigma-Aldrich	34871	≥99.9
2-propanol	67–63–0	Sigma-Aldrich	34863	99.9
1-butanol	71–36–3	Sigma-Aldrich	34867	≥99.7
2-butanol	78–92–2	Sigma-Aldrich	B85919	≥99
dimethyl sulfoxide	67–68–5	Sigma-Aldrich	34869	≥99.7
purified air	132259–10–10	Air Liquide	CA-1001–00762	100
hydrogen	1333–74–0	Airgas	HY UHP200	99.999
methane	74–82–8	Airgas	ME UHP200	99.99
nitrogen	7727–37–9	Airgas	NI UHP200	99.999

### Methods and Theory of Inverse Gas Chromatography

The IGC method is based on the measurement of retention times of probe gases or vapors injected into a column containing the sample material. The retention time is dependent on the interaction of the probe with the sample. The retention time is also dependent on the flow rate and the path to the detector. Therefore, the retention time of a probe must be corrected for by using “dead time”, the time it takes for a noninteracting probe to reach the detector. Methane was used to approximate the dead time of the system, as methane may interact weakly with the film. Pulses of VOC probes were passed through a 2D film cell module containing a thin film of Sylgard 184, and the retention times of the probes were measured using a flame-ionization detector. The measured retention time is determined by the peak max of the elution signal minus the dead time of the system. The volume of carrier gas required to elute the probe from the column is defined as the retention volume. Retention volume is calculated using the following equation

1 $$V_{n}=\frac{j}{m}\cdot F\cdot t_{\mathrm{retention}}\cdot \frac{T}{298.15\;K}$$

where, *V*_n_ is the net retention volume, *j* is the pressure gradient correction from James-Martin factor that accounts for the compressibility of the flowing gas and the pressure drop along the bed, *m* is mass of the sample, *F* is the exit flow rate, *t*_retention_ is the retention time of the probe species minus the retention time of methane, and *T* is column temperature.^7^ Generally, the net retention volume contains the summation of all probe–sample interactions. This can be expressed using the following equation

2 $$V_{n}=K_{L}V_{L}+K_{\mathrm{GL}}S_{\mathrm{GL}}+K_{L}K_{\mathrm{LS}}S_{\mathrm{LS}}$$

where *V*_n_ is the net retention volume, *K*_L_ is the absorption partition coefficient, or Henry’s solubility coefficient, *V*_L_ is the volume of the liquid (polymer) phase, *K*_GL_ is the adsorption partition coefficient at an interface of the gas and liquid, *S*_GL_ is the surface area of the gas–solid interface, *K*_LS_ is the partition coefficient of the liquid–solid interface, and *S*_LS_ is the surface area of the liquid–solid interface.^5^ When *K*_L_ ≫ *K*_GL_ and *K*_L_ ≫ *K*_LS_, *V*_n_ ≈ *K*_L_, including adjustments to the volumes and surface areas as part of these inequalities, so using a simple conversion the Henry’s solubility coefficient can be calculated by

3 $$H=V_{n}\rho$$

where *H* is the solubility coefficient, *V*_n_ is the net retention volume, and ρ is the density of the polymer.^5^ *H* is dimensionless. After eq 1 net retention volume has units of cm3/g. Henry’s law requires the amount of dissolved gas in a liquid phase to be linearly proportional to the partial pressure in the gas phase. This is upheld under infinite dilution conditions (probe–probe interactions are negligible).

Determination of diffusion coefficients using 2D IGC uses Fick’s Law of Diffusion and the van Deemter equation. Fickian diffusion describes the process by which particles spread from areas of higher concentration to areas of lower concentration, driven by a concentration gradient. We assume that diffusion occurs one-dimensionally, normal to the film; the film is treated as an infinite slab. The van Deemter equation is described by

4 $$\mathrm{HETP}=A+\frac{B}{u_{z}}+Cu_{z}$$

where HETP is the height equivalent to a theoretical plate, *u*_*z*_ is the linear velocity of the mobile phase, and *A*, *B*, and *C* are constants.^6^ The eddy diffusion term, *A*, accounts for the multiple flow paths that a gas molecule can take through the column. Due to the random nature of these paths, some molecules travel faster while others travel slower, leading to peak broadening. This term is independent of the linear velocity and is influenced by the geometry of the packing material. A less tortuous path reduces eddy diffusion effects, leading to narrower peaks. Longitudinal diffusion term, *B*/*u*_*z*_, accounts for the natural tendency of gas molecules to diffuse along the length of the column. This diffusion occurs because of the concentration gradient inherent to a pulse. This term is inversely proportional to the linear velocity. The mass transfer term, *Cu*_*z*_, accounts for the resistance to mass transfer between the mobile phase (vapor species) and the stationary phase (thin film). This resistance causes a delay in the equilibrium between the two phases, leading to peak broadening. This term is directly proportional to the linear velocity. Linear velocity is calculated as the quotient of volumetric flow rate and the channel area normal to the flow. The peak variance, the square of the standard deviation of the peak, is used to calculate diffusion coefficients. HETP is experimentally determined from the peak variance using eq 5

5 $$\mathrm{HETP}=Z\left(\frac{\sigma^{2}}{t_{\mathrm{dead}}^{2}}\right)$$

where *Z* is the length dimension of the column, σ^2^ is the peak variance.^5,31^ σ is calculated using the full width at half-maximum divided by 2.35. At high linear velocity, longitudinal diffusion is negligible. In this case, the van Deemter equation can be simplified to

6 $$\mathrm{HETP}=A+Cu_{z}$$

because *B*/*u*_*z*_ ≈ 0. By varying the flow rate and therefore linear velocity, *C* and *A* can be determined from a linear fit of HETP and linear velocity. An example of the linear fit to the HETP vs linear velocity is presented in Figure 2 for toluene. A high r-squared value assures linearity of the data included in the fit. Huang et al.^9^ reports an equation relating the peak variance to the linear velocity and diffusion coefficient for the geometry of a rectangular column in the IGC. *The authors* use a dimensionless peak variance in their derivation, analogous to the product HETP and the governing dimension length. We prefer to present the solution to include *C* from the van Deemter equation as

7 $$D=\frac{{2\tau }^{3}H}{3\mathrm{hC}}$$

where *D* is the diffusion coefficient, τ is the thickness of the polymer, *H* is the Henry’s solubility coefficient, *h* is the height of the column, and *C* is determined from the slope of the linear fit.^9^

**Figure 2:**
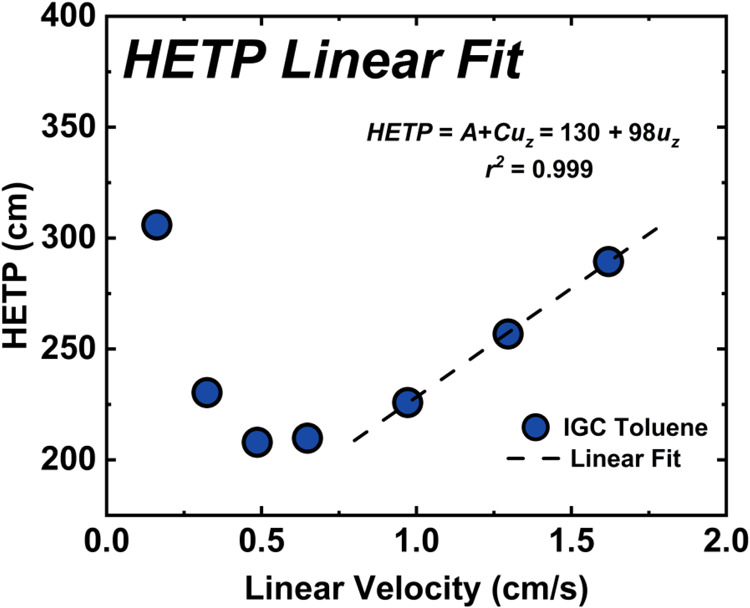
Height equivalent to a theoretical plate (HETP) as a function of linear velocity (*u*_*z*_) of toluene and Sylgard 184 as measured in two-dimensional (2D) inverse gas chromatography (IGC). Dashed line is the linear fit at high linear velocities of HETP vs *u*_*z*_.

## Results

### Validation of Infinite Dilution and Absorption-Dominated Conditions

Accurate Henry’s coefficient determination requires two conditions: (1) infinite dilution (negligible probe–probe interactions) and (2) absorption-dominated sorption (*K*_L_ ≫ *K*_LS_, *K*_L_ ≫ *K*_GL_). Infinite dilution was experimentally verified by lowering the probe concentration until the retention time and peak shape did not change. Generally, soft rubbery polymers, including PDMS, at temperatures above the glass transition temperature can be treated as having negligible adsorption effects so we assume we can ignore this contribution in reference to the external surface adsorption and not adsorption on the filler particles.^32^

Measured Henry’s solubility coefficients for 29 VOCs in Sylgard 184 at 20 °C are presented in Table 2, both in dimensionless form (*H*) and normalized to saturation pressure (*H*/*P*_sat_) to facilitate temperature-independent comparisons. Most of the temperature dependence of the partition coefficient is driven by heats of condensation, which are captured by saturation pressure. Measurement uncertainties represent two standard deviations from at least three replicate injections. Literature values for pure PDMS polymers at 22.5–30 °C are included for comparison. The normalization *H*/*P*_sat_ enables direct comparison across temperatures by accounting for vapor pressure differences.

**Table 2:** Measured Henry’s Solubility Coefficient for Sylgard 184 Thin Film at 20 °C in Dimensionless Form (*H*) and Normalized to the Saturation Pressure at Temperature of Measurement (*H*/*P*_sat_) for Comparison across Temperatures^a^

compound	*H*	measurement error %	*H*/*P*_sat_ (1/kPa)	*H*/*P*_sat_ (1/kPa) literature values for PDMS at 22.5 to 30 °C
*n*-hexane	277	2.8	17.1	17.3,^33^ 10.5,^34^ 10.4 ^35^
2,3-dimethylbutane	178	3.6	7.81	-
2-methylpentane	193	3.4	7.57	5.33 ^35^
*n*-heptane	804	2.4	171	132,^33^ 96.3,^34^ 97.3 ^35^
*n*-octane	2060	3.8	1470	1180,^33^ 842,^34^ 871 ^35^
isooctane	692	3.0	136	-
*n*-nonane	7460	0.4	1.77 × 10^4^	9130 ^33^
*n*-decane	1.90 × 10^4^	0.9	1.50 × 10^5^	6.02 × 10^4^
*n*-undecane	5.10 × 10^4^	1.4	1.36 × 10^6^	-
cyclohexane	522	1.1	50.3	40.4,^33^ 36.3 ^34^
cyclooctane	6515	1.5	5.41 × 10^5^	-
toluene	1476	1.2	504	315,^33^ 267.4,^34^ 92.5,^35^ 301.0 ^36^
o-xylene	5148	0.6	7860	4180 ^33^
dichloromethane	130	4.3	2.73	1.58 ^34^
chloroform	297	3.3	14.1	9.94,^33^ 8.37 ^34^
carbon tetrachloride	517	1.3	42.6	29.6,^33^ 23.5 ^34^
acetonitrile	142	3.3	15.2	<2.54 ^33^
diethyl ether	118	4.8	2.14	1.35 ^33^
acetone	181	2.6	7.33	2.93,^33^ 3.71,^37^ 4.39 ^38^
methylethyl ketone	389	3.9	40.2	20.4 ^37^
ethyl acetate	433	3.1	44.7	21.7 ^33^
butyl acetate	1214	1.7	1110	-
methanol	171	4.3	13.3	-
ethanol	235	4.9	39.8	22.7,^33^ 25.7 ^39^
1-propanol	559	4.0	277	89.0,^33^ 64.8 ^40^
2-propanol	269	4.0	63.8	17.2,^33^ 17.8 ^40^
1-butanol	1484	2.1	1680	644 ^33^
2-butanol	653	1.7	391	114 ^38^
dimethyl sulfoxide	3590	1.1	6.41 × 10^4^	-

a Measurement error calculated from two-standard deviations

Diffusion coefficients extracted via eq 7 are presented in Table 3 alongside *r*2 values from HETP vs u_z linear fits and available literature data for PDMS. All *r* ^2^ values exceed 0.97 (except acetone, *r*2 = 0.94), confirming measurements were conducted in the appropriate high-velocity regime where longitudinal diffusion contributions are negligible. There are fewer diffusion measurements on PDMS materials available in literature. Those that are near room temperature are presented. Other measurements, conducted at significantly higher temperatures exist for VOCs in PDMS, but we do not find it useful to directly compare these values. Instead, we will use the Arrhenius formula with a determined apparent activation energy of diffusion and a diffusion pre-exponential factor determined by diffusion measurements at higher temperatures to extrapolate down to 20 °C, presented in eq 8.

8 $$\ln\left(D\right)=-\frac{E_{D}}{\mathrm{RT}}+\ln\left(D_{0}\right)$$

where *D* is the diffusion coefficient at temperature, *E*_*D*_ is an apparent activation energy of diffusion, *R* is the ideal gas constant, *T* is temperature, and *D*_0_ is a diffusion pre-exponential factor. Calculated diffusivity values retrieved from literature are presented in Table 3 with an asterisk (*).

**Table 3:** Measured Diffusion Coefficients (*D*) for Sylgard 184 Thin Film at 20 °C^a^

compound	*D* × 10^–5^ (cm^2^/s)	*r* ^*2*^	*D* × 10^–5^ (cm^2^/s) literature values
*n*-hexane	2.68	0.994	-
2,3-dimethylbutane	1.52	0.972	-
2-methylpentane	2.55	0.991	-
*n*-heptane	1.84	0.999	3.6, 4.2 [20 °C*][Bibr ref14]
*n*-octane	1.10	0.998	1.4, 1.6 [20 °C*][Bibr ref14]
isooctane	1.04	0.970	-
*n*-nonane	0.82	0.993	0.56, 0.71 [20 °C*][Bibr ref14]
*n*-decane	0.28	0.996	-
*n*-undecane	0.10	0.999	-
cyclohexane	1.80	0.999	-
cyclooctane	0.57	0.998	-
toluene	2.04	0.999	2.4, 2.8 [20 °C*],^14^ 0.115 [25 °C],^41^ 0.18 [25 °C],^42^ 0.035–0.04 [25 °C][Bibr ref43]
o-xylene	0.94	0.999	-
dichloromethane	7.84	0.996	-
chloroform	3.37	0.999	0.651 [40 °C][Bibr ref44]
carbon tetrachloride	1.91	0.999	-
acetonitrile	8.94	0.998	-
diethyl ether	3.39	0.998	-
acetone	5.73	0.940	-
methylethyl ketone	1.95	0.999	-
ethyl acetate	1.89	0.995	-
butyl acetate	1.54	0.999	-
methanol	8.25	0.999	1.870 [40 °C][Bibr ref44]
ethanol	2.20	0.981	0.152 [25 °C][Bibr ref39]
1-propanol	1.30	0.999	-
2-propanol	1.39	0.980	-
1-butanol	0.93	0.998	0.311 [40 °C][Bibr ref44]
2-butanol	1.08	0.997	0.225 [40 °C][Bibr ref44]
dimethyl sulfoxide	1.01	0.981	-

a R-Squared (*r* ^*2*^) from a Linear Fit of HETP vs linear velocity. Asterisk (*) denotes that the literature value was predicted from higher temperature data using an Arrhenius fit

Diffusion coefficients range from 1.0 × 10^–6^ cm^2^/s (n-undecane) to 8.9 × 10^–5^ cm^2^/s (acetonitrile), spanning nearly 2 orders of magnitude. Several clear trends emerge: (1) *D* decreases with increasing molecular size (molecular weight), as larger molecules experience greater steric hindrance navigating the polymer network; (2) linear alkanes show systematic decreases in *D* with chain length; (3) small polar molecules (acetonitrile, methanol, dichloromethane) exhibit the highest diffusivities; (4) branched isomers diffuse slightly slower than linear analogs of equal molecular weight due to increased steric bulk.

## Discussion

### Comparison with Pure PDMS: Role of Silica Filler

Sylgard 184 and pure PDMS share the same fundamental polymer backbone (cross-linked dimethylsiloxane chains) but differ substantially in composition due to Sylgard 184’s silica filler content (30–60 wt %). Silica (ρ ≈ 2.2 g/cm^3^) is approximately twice as dense as PDMS (ρ ≈ 0.97 g/cm^3^). For our measured Sylgard 184 density of 1.05 g/cm^3^, we estimate the composite contains ∼18 vol % silica and ∼82 vol % PDMS. If the silanized silica were completely inert (noninteracting), we would expect solubility coefficients in Sylgard 184 to be ∼18% lower than pure PDMS due to reduced polymer volume available for VOC absorption. However, Table 2 reveals the opposite trend: solubility coefficients for Sylgard 184 systematically exceed those of pure PDMS, often substantially. This enhanced solubility indicates that the silica filler actively participates in VOC sorption rather than merely occupying inert volume. The surface-modified silica provides additional binding sites and interaction opportunities beyond those available in the PDMS matrix alone. While the silica surface is treated with hexamethyldisilazane to generate predominantly dimethylvinylated and trimethylated surface groups (reducing hydrophilicity and improving PDMS compatibility), complete surface coverage is unlikely. Residual silanol (Si–OH) groups inevitably remain, offering hydrogen-bonding sites absent in the pure PDMS backbone.

This interpretation is strongly supported by the alcohol data. Excluding alcohols and a few outliers (*n*-decane, ethyl acetate, acetonitrile), Sylgard 184 solubility coefficients fall within a factor of 2 of pure PDMS literature values (15 of 21 compounds). Some scatter in this comparison likely reflects inconsistency among the literature PDMS values themselves, which derive from different studies using varied PDMS formulations, cross-link densities, and measurement methods. In contrast, alcohol solubilities show dramatic enhancements: methanol (not reported for pure PDMS), ethanol (1.75× higher), 1-propanol (3.1× higher), 2-propanol (3.7× higher), 1-butanol (2.6× higher), and 2-butanol (3.4× higher). These systematic deviations implicate hydrogen-bonding interactions with residual silanol groups on the silica filler surface as the primary mechanism for enhanced alcohol solubility. For non-hydrogen-bonding VOCs (alkanes, aromatics), the silica filler may play competing roles of removing volume and additional van der Waals interactions at the filler–polymer interface and with the organosilane surface groups themselves.

### Molecular Determinants of Diffusion in Filled PDMS

Our diffusion measurements reveal several systematic trends that illuminate how molecular properties and filler presence govern transport through Sylgard 184. The inverse relationship between diffusivity and molecular weight is well-established in polymer physics. Larger molecules experience greater frictional resistance and steric hindrance from the polymer network. For the *n*-alkane series (hexane through undecane), log­(*D*) decreases approximately linearly with carbon number, similar to Graham’s law, where the rate of diffusion or effusion of a gas is inversely proportional to the square root of its molar mass. Branched isomers (isooctane vs *n*-octane, 2-methylpentane vs *n*-hexane) diffuse slightly slower than their linear counterparts despite similar molecular weights, reflecting increased steric bulk that impedes hopping between free-volume pockets. Small polar molecules (acetonitrile, methanol, dichloromethane) exhibit the highest diffusivities (5.7–8.9 × 10^–5^ cm^2^/s). These high diffusivities are coupled with combination of high *D* coupled with moderate-to-high H has important implications for permeability (*P* = *D* × *H*), as both factors contribute multiplicatively. Comparison with pure PDMS literature data shows reasonable agreement (within 1 order of magnitude), though direct comparison is complicated by differences in temperature, cross-link density, and the presence of silica filler in Sylgard 184. For *n*-alkanes where temperature-dependent data exist,^14^ extrapolating literature values to 20 °C using Arrhenius parameters yields excellent agreement, with all measured diffusion coefficients falling within a factor of 2 of the extrapolated literature values: *n*-heptane (1.84 vs 3.6, 4.2 × 10^–5^ cm^2^/s), *n*-octane (1.10 vs 1.4, 1.6 × 10^–5^ cm^2^/s), *n*-nonane (0.82 vs 0.56, 0.71× 10^–5^ cm^2^/s), and toluene (2.04 vs 2.4, 2.8 × 10^–5^ cm^2^/s). The slight tendency toward lower *D* in Sylgard 184 might reflect tortuosity effects from the silica filler. Particles create more tortuous diffusion pathways compared to neat polymer. However, these effects are typically modest with filler at a low loading level, 18 vol % estimated. Thus, we suspect this discrepancy arises from different measurement techniques and sample preparation methods rather than fundamental differences in alkane-PDMS interactions. Our IGC measurements at infinite dilution probe single-molecule diffusion, whereas some literature techniques (e.g., time-lag methods) may inadvertently operate at higher concentrations where polymer plasticization or concentration-dependent diffusivity could affect results.

### Implications for VOC Permeation and Membrane Applications

The fundamental relationship *P* = *D* × *H* dictates that both solubility and diffusivity contribute multiplicatively to overall permeability. Our data reveals that different VOC classes achieve high permeability through different mechanisms. Small polar molecules (acetonitrile, methanol, dichloromethane) have high permeability driven primarily by high diffusivity (*D* = 5–9 × 10^–5^ cm^2^/s) despite moderate solubility. Medium-chain alkanes and aromatics (*n*-octane, toluene, o-xylene) display balanced contributions from moderate-to-high *H* (10^2^–10^4^) and moderate *D* (0.8–2.0 × 10^–5^ cm^2^/s). Long-chain alkanes, larger alcohols, and dimethyl sulfoxide (*n*-decane, n-undecane, 1-butanol, DMSO) have high permeability driven by very high solubility (*H* > 10^4^) despite low diffusivity (*D* = 0.1–1.5 × 10^–5^ cm^2^/s). Sylgard 184’s enhanced alcohol solubility (relative to pure PDMS) could be advantageous for applications where selective alcohol permeation is desired. Conversely, for protective cushion or barrier applications, the elevated alcohol permeability represents a potential vulnerability.

### Advantages and Limitations of 2D IGC Methodology

This study demonstrates several advantages of the 2D thin-film IGC approach over traditional packed-column methods. Most critically, film thickness is directly and accurately measured (0.064 ± 0.002 mm), eliminating the largest uncertainty source in diffusivity calculations. Flow streamlines are well-defined and mass transfer boundary conditions are unambiguous, unlike the complex interstitial spaces in packed beds. The absence of support particles eliminates substrate-related artifacts (e.g., surface adsorption on support material).

However, limitations exist. In our setup, the 2D column lacks active temperature control, restricting measurements to ambient conditions unless placed in a temperature-controlled enclosure. A large oven would be required for finer temperature control. In this study, our multipoint thickness measurements confirm excellent uniformity (±3%) in the sample. Finally, very high or very low diffusivities may fall outside the experimentally accessible velocity range where the van Deemter simplified equation applies, whereas, ultrafine particles in packed beds may shorten the determinant distances.

### Broader Context and Future Directions

This work adds to the limited but growing literature applying 2D IGC to polymer thin films.^7–9^ While traditional packed-column IGC remains more common, thin-film approaches offer compelling advantages for fundamental studies where accuracy in D is paramount. Our results provide valuable thermodynamic and transport data for VOC-Sylgard 184 interactions. This data is directly applicable to predicting permeation, designing separations, assessing associated chemical compatibility risks, and modeling environmental exposures.

Future work should explore temperature-dependent measurements to extract diffusivity activation energies and enthalpies of sorption, enabling predictions across application-relevant temperature ranges. Systematic variation of silica filler content would deconvolve filler effects from intrinsic PDMS behavior. Extension to mixed VOC-water systems would address potential competitive sorption or synergistic effects in real-world multicomponent exposures. Finally, comparison with other filled PDMS formulations would establish whether our findings generalize or are specific to the polymer’s particular composition.

## Conclusions

This study demonstrates the utility of 2D thin-film IGC for simultaneous measurement of Henry’s solubility and Fickian diffusion coefficients in polymer thin films. The methodology offers some advantages over traditional packed-column approaches. Most notably, it provides the ability to precisely measure and control film thickness, which is the critical length scale for accurate diffusivity determination. We measured these properties for 29 volatile organic compounds in Sylgard 184 at 20 °C, providing a data set spanning diverse chemical functionalities. Comparison with pure PDMS literature data reveals that the polymer’s silica filler content actively participates in VOC sorption, enhancing solubility (particularly for hydrogen-bonding species such as alcohols) through interactions with residual surface hydroxyl groups. Diffusion coefficients range from 1.0 × 10^–6^ cm^2^/s (*n*-undecane) to 8.9 × 10^–5^ cm^2^/s (acetonitrile) and align well with literature values for pure PDMS, indicating the silica filler introduces only modest tortuosity effects at this loading level. These results provide essential data for predicting VOC permeation in Sylgard 184 and enhance our understanding of how polymer composite composition influences vapor sorption and transport.

We considered directly addressing whether the thin-film or traditional packed-column approach is better, but as our experience with IGC has grown, we have found that the best technique is the one that best suits the material. Cryomilling into a powder works well for some polymers, others require a very thin coating on a traditional support, and polymers that are easily poured or cast can perform well as thin films. The choice is best made on a case-by-case basis, and with thorough characterization any of these preparation techniques is valid. A comprehensive comparison of these approaches is beyond the scope of the present study and is a direction for future work.
